# *Enterococcus faecalis* Sex Pheromone cCF10 Enhances Conjugative Plasmid Transfer *In Vivo*

**DOI:** 10.1128/mBio.00037-18

**Published:** 2018-02-13

**Authors:** Helmut Hirt, Kerryl E. Greenwood-Quaintance, Melissa J. Karau, Lisa M. Till, Purna C. Kashyap, Robin Patel, Gary M. Dunny

**Affiliations:** a Department of Microbiology and Immunology, Medical School, University of Minnesota, Minneapolis, Minnesota, USA; b Division of Clinical Microbiology, Department of Laboratory Medicine and Pathology, Mayo Clinic, Rochester, Minnesota, USA; c Division of Gastroenterology and Hepatology, Department of Medicine, Mayo Clinic, Rochester, Minnesota, USA; d Division of Infectious Diseases, Department of Medicine, Mayo Clinic, Rochester, Minnesota, USA; University of Washington

**Keywords:** antibiotic resistance transfer, cell-cell signaling, competitive fitness, gut microbiota, mobile genetic element

## Abstract

Cell-cell communication mediated by peptide pheromones (cCF10 [CF]) is essential for high-frequency plasmid transfer *in vitro* in *Enterococcus faecalis*. To examine the role of pheromone signaling *in vivo*, we established either a CF-producing (CF+) recipient or a recipient producing a biologically inactive variant of CF (CF− recipient) in a germfree mouse model 3 days before donor inoculation and determined transfer frequencies of the pheromone-inducible plasmid pCF10. Plasmid transfer was detected in the upper and middle sections of the intestinal tract 5 h after donor inoculation and was highly efficient in the absence of antibiotic selection. The transconjugant/donor ratio reached a maximum level approaching 1 on day 4 in the upper intestinal tract. Plasmid transfer was significantly lower with the CF− recipient. While rescue of the CF− mating defect by coculture with CF+ recipients is easily accomplished *in vitro*, no extracellular complementation occurred *in vivo*. This suggests that most pheromone signaling in the gut occurs between recipient and donor cells in very close proximity. Plasmid-bearing cells (donors plus transconjugants) steadily increased in the population from 0.1% after donor inoculation to about 10% at the conclusion of the experiments. This suggests a selective advantage of pCF10 carriage distinct from antibiotic resistance or bacteriocin production. Our results demonstrate that pheromone signaling is required for efficient pCF10 transfer *in vivo*. In the absence of CF+ recipients, a low level of transfer to CF− recipients occurred in the gut. This may result from low-level host-mediated induction of the donors in the gastrointestinal (GI) tract, similar to that previously observed in serum.

## IMPORTANCE

Horizontal gene transfer is a major factor in the biology of *Enterococcus faecalis*, an important nosocomial pathogen. Previous studies showing efficient conjugative plasmid transfer in the gastrointestinal (GI) tracts of experimental animals did not examine how the enterococcal sex pheromone response impacts the efficiency of transfer. Our study demonstrates for the first time pheromone-enhanced, high-frequency plasmid transfer of *E. faecalis* plasmid pCF10 in a mouse model in the absence of antibiotic or bacteriocin selection. Pheromone production by recipients dramatically increased plasmid transfer in germfree mice colonized initially with recipients, followed by donors. The presence of a coresident community of common gut microbes did not significantly reduce *in vivo* plasmid transfer between enterococcal donors and recipients. In mice colonized with enterococcal recipients, we detected plasmid transfer in the intestinal tract within 5 h of addition of donors, before transconjugants could be cultured from feces. Surprisingly, pCF10 carriage provided a competitive fitness advantage unrelated to antibiotic resistance or bacteriocin production.

## INTRODUCTION

Horizontal gene transfer by conjugation is a major contributor to genome plasticity and evolution in *Enterococcus faecalis* (1). This Gram-positive intestinal commensal bacterium is also a major nosocomial pathogen, with infections often following dysbiosis triggered by antimicrobial therapy. *E. faecalis* has high intrinsic resistance to several antibiotics, and a multitude of acquired antibiotic resistance genes on mobile genetic elements further complicate antimicrobial therapy (2). Determination of the genome sequence of V583, the first vancomycin-resistant enterococcal isolate in North America (3), provided a paramount example of the propensity of this organism to acquire multiple mobile elements in the clinical setting, including conjugative pheromone-responsive plasmids (4).

**FIG 1:**
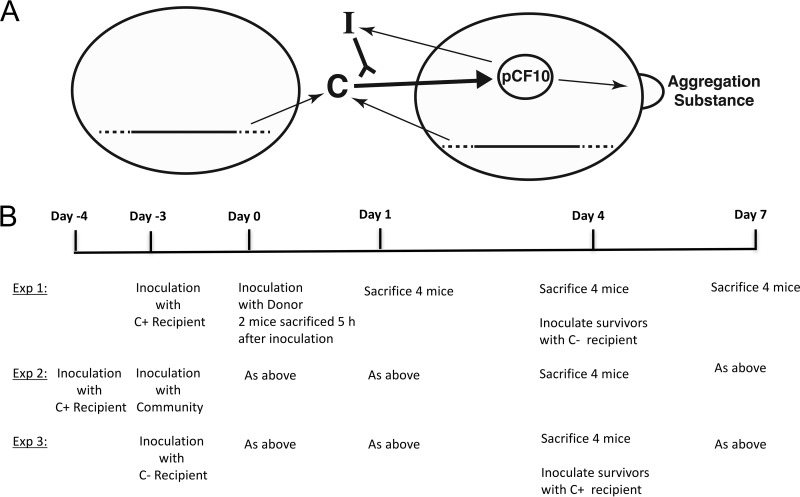
Role of sex pheromone cCF10 in transfer of pCF10 in the mouse intestinal tract. (A) Pheromone signaling between recipients (left) and donors (right). Pheromone cCF10 (CF) (shown as **C** in the figure) (sequence LVTLVFV) is a product of the *ccfA* gene found in the chromosome (horizontal black line with dashed ends in both cells) of all *E. faecalis* strains. Import of CF into the donor cell induces expression of aggregation substance and other pCF10 gene products required for pCF10 transfer. The inhibitor peptide iCF10 (shown as **I** in the figure) (sequence AITLIFI) encoded by the *prgQ* gene of pCF10, is a competitive inhibitor of the conjugation-inducing activity of CF. See reference 5 for more details. (B) Timeline for three *in vivo* conjugation experiments described in this paper. A total of 14 animals in four separate cages (four mice/cage) were inoculated with recipient cells before introduction of the donor strain, OG1Sp(pCF10). For experiments 1 and 2, a CF+ recipient initially established, while in experiment 3, a CF− recipient was first established. For all three experiments, two mice were sacrificed 5 h after the inoculation with the donor, the isolated intestine was divided into three sections, and donors, recipients, and transconjugants were enumerated by plating on selective media. Four mice from one cage were sacrificed on days 1, 4, and 7 following donor inoculation, and enterococci associated with intestinal tissue were enumerated. Fecal samples were taken for all individual animals on days 1, 4, and 7. For experiment 2 with the competing microbial community, the CF+ recipient strain was inoculated on day −4, with a defined multispecies community inoculated on day −3, and donors on day 0.

Pheromone-responsive plasmids in *E. faecalis* are the most extensively characterized conjugative plasmids among Gram-positive bacteria. The complex regulatory circuits controlling transfer of the pheromone-responsive plasmids pAD1 and pCF10 have been the major focus of these studies (5). Pheromone-responsive plasmids carry genes that can encode antibiotic resistance and hemolysin/bacteriocin production, but these plasmids can also be devoid of genes encoding detectable phenotypic markers (6, 7). Conjugative transfer of the tetracycline resistance plasmid pCF10 is initiated in the donor by sensing cCF10 (CF), a 7-amino-acid hydrophobic peptide encoded within the chromosome. A pCF10-encoded inhibitory peptide iCF10 (iCF) modulates the pheromone response by direct competition with CF (Fig. 1A); the induction state of an individual donor cell depends on the intracellular molar ratio of the two peptides (5). Expression of aggregation substance and other proteins on the cell surface of the donor follows pheromone uptake, with subsequent formation of macroscopic aggregates and plasmid transfer at an efficiency of up to 10^−1^ transconjugants/donor (T/D) in liquid medium *in vitro*. In donors carrying pCF10, a low level of induction (in the absence of recipients) can also be triggered by host factors in serum, via a mechanism involving differential sequestration of donor-produced iCF and CF peptides (8, 9).

While the pheromone response has been well characterized *in vitro*, the role of this form of cell-cell communication in the natural habitat of *E. faecalis* has not been determined. Efficient transfer of pheromone-responsive plasmids in the gastrointestinal (GI) tracts of hamsters and pigs following antibiotic-induced suppression of the normal gut microbiota has been observed (10, 11). More recently, transfer was demonstrated in conventional mice following long-term administration of donors and recipients in drinking water to enhance stable colonization (12). None of these studies directly examined the effects of pheromone signaling on transfer, and interpretation of the results was affected by either pretreatment of animals with antibiotics, by the use of plasmids encoding bacteriocin production (which could select against plasmid-free cells), or both.

Our goal was to answer several basic questions related to plasmid transfer of pCF10 *in vivo*. We established a germfree mouse model to investigate the kinetics and localization of pCF10 plasmid transfer without interference by resident microbiota or antibiotics. Plasmid transfer in the intestinal tract was detected a few hours after donor inoculation in the intestinal tract, before either donors or transconjugants appeared in feces. Introduction of a competing defined microflora did not impact plasmid transfer. Plasmid transfer also occurred in the absence of pheromone production by the recipient, but at significantly lower rates, suggesting a basal level of donor induction by the host. Efficient transfer into recipients producing a biologically inactive variant of CF (CF− recipients) was not restored in the intestine by coresident CF-producing recipients (CF+ recipients), suggesting that natural pheromone signaling occurred in microniches where mating cells were colocalized. The data also suggest that pCF10 may contribute to competitive fitness of its host cell in the gut by an unknown mechanism.

## RESULTS

### Efficient plasmid transfer in the germfree mouse.

An overview and summary of the experimental setup and time line are depicted in Fig. 1B. These protocols were based on preliminary experiments analogous to experiments 1 and 3 below, and where very similar results were obtained (see Tables S1 and S2 in the supplemental material). All mice were initially monocolonized with recipients that were allowed to establish for 3 or 4 days prior to addition of donors. In experiment 2, mice were inoculated with a defined microbial community in the interim between inoculation of recipients and donors. Preliminary experiments indicated that plasmid transfer could be detected by plating feces 1 day after donor inoculation, but not 5 h after donor inoculation (Table S1). On the basis of these results, we plated both feces and homogenized intestinal tissue segments 1, 4, and 7 days after donor inoculation. In addition, we sacrificed and plated homogenized intestinal segments from two mice 5 h after donor inoculation. This enabled us to assess plasmid transfer in the intestinal tract at an early time point when feces were still negative for transconjugants, confirming transfer within the GI tract as opposed to transfer in expelled feces and subsequent coprophagic reingestion.

10.1128/mBio.00037-18.4 TABLE S1  Numbers of recipients, donors, and transconjugants in feces. Fecal samples were obtained from 12 mice in a preliminary experiment. The donor strain was OG1Sp(pCF10), and the recipient strain was OG1RF. Download TABLE S1, DOCX file, 0.01 MB. Copyright © 2018 Hirt et al. 2018 Hirt et al. This content is distributed under the terms of the Creative Commons Attribution 4.0 International license.

10.1128/mBio.00037-18.5 TABLE S2  T/D ratios in two preliminary experiments with wild-type (CF+ [C+]) and cCF10-deficient mutant (CF− [C−]) as the established recipient in the germfree mouse model. In the CF− experiment, the mice are inoculated with the C+ recipient on day 4. The T/D ratios are averages ± standard deviations for four mice. The T/D ratios in the upper, middle, and lower sections of the intestine and in feces are shown. Download TABLE S2, DOCX file, 0.01 MB. Copyright © 2018 Hirt et al. 2018 Hirt et al. This content is distributed under the terms of the Creative Commons Attribution 4.0 International license.

**FIG 2:**
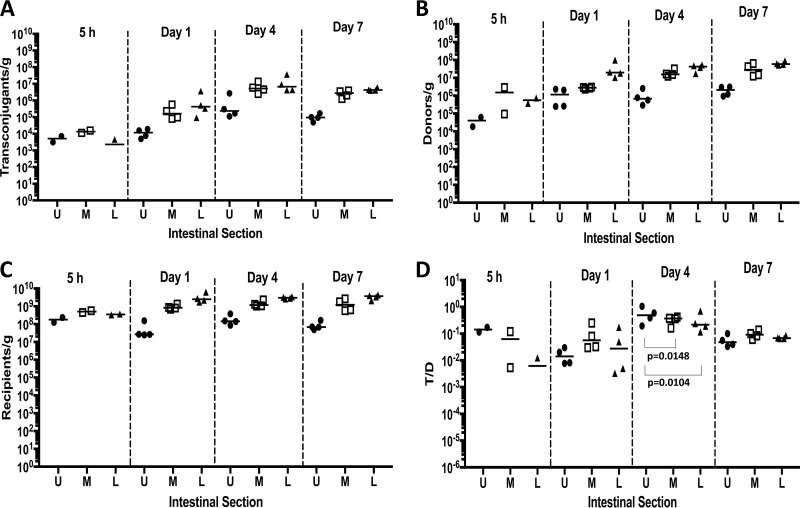
Plasmid transfer in the germfree mouse model, wild-type donor and recipient (experiment 1; Fig. 1B). (A to C) Transconjugants (A), donors (B), and recipients (C) are shown as CFU/gram of intestinal tissue, as enumerated on selective agar medium. (D) The frequency of plasmid transfer is shown as the transconjugant/donor (T/D) ratio. On day 4, the T/D ratio is significantly higher in the upper intestinal tract. Values for the upper (U), middle (M), and lower (L) sections of the intestinal tract are shown. Each symbol represents the value for a section from an individual mouse as follows: ●, upper section; □, middle section; ▲, lower section. The bars represent the median values for the intestinal sections.

Experiment 1 used wild-type recipient and wild-type donors. Recipients, donors, and transconjugants established at the highest cell density in the lower intestinal tract, whereas the upper intestinal tract supported the lowest bacterial populations as expected (Fig. 2A to C). Transconjugants were detected in the upper and middle portions of the intestinal tract 5 h after donor inoculation in both mice, while only one mouse was positive for transconjugants in the lower section of the intestinal tract. The population of transconjugants rose during the experiment (Fig. 2A). The number of recipients remained at nearly constant levels following addition of donors (Fig. 2C), while donors steadily increased in all sections of the intestinal tract over time (Fig. 2B). When we assessed the efficiency of plasmid transfer by enumerating the transconjugants per donor (T/D) ratio (Fig. 2D), we detected T/D ratios in the intestinal tracts between 10^−2^ to 10^−1^ 5 h after donor inoculation. These ratios remained at that level in all sections of the intestinal tract throughout the experiment, suggesting that even at the low densities found at the earliest time point, the donor populations had good access to recipients. A significantly higher T/D ratio was observed on day 4 in the upper intestinal tract (*P* = 0.0148 for upper and middle sections; *P* = 0.0104 for upper and lower sections), whereas the difference in the T/D ratio for the middle and lower sections of the intestinal tract was not significant.

**FIG 3:**
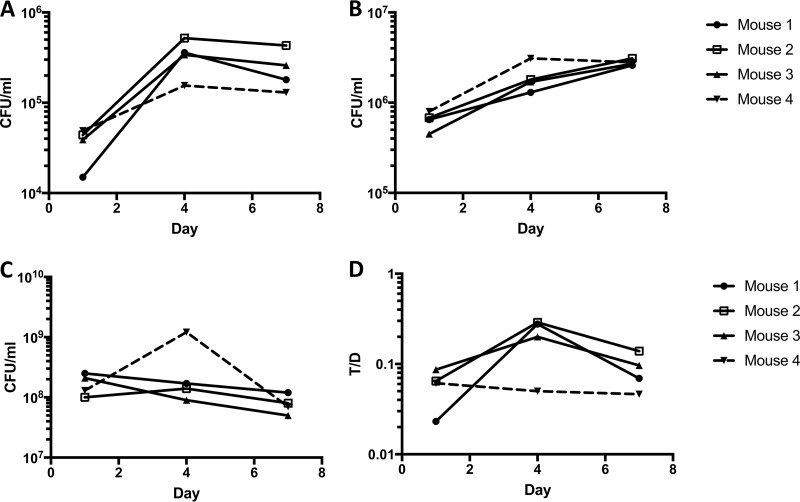
Time course of colonization and plasmid transfer as enumerated from feces (experiment 1; Fig. 1B). (A to C) Cell numbers of transconjugants (A), donors (B), and recipients (C) over the course of the experiment. The results for one cage with four mice (sacrificed on day 7) are shown. (D) Plasmid transfer expressed as a ratio of transconjugants/donor (T/D).

**FIG 4:**
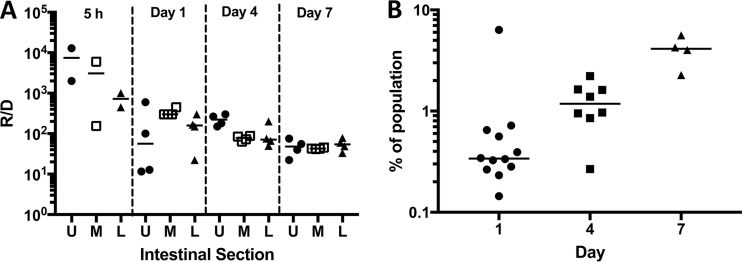
Population shift in favor of pCF10-carrying cells (experiment 1; Fig. 1B). (A) Recipient-to-donor (R/D) ratio over the time course of the experiment. Each symbol represents the value for a section from an individual mouse, and the bars represent the median values for the intestinal sections. The upper (U) (●), middle (M) (□), and lower (L) (▲) sections of the intestinal tract are shown. (B) Percentage of cells harboring pCF10 (donors plus transconjugants) in feces over time.

Cell numbers determined for donors, recipients, and transconjugants collected from feces mirrored trends seen in intestinal tract sections. At later time points, recipient populations decreased slightly, while both donors and transconjugants increased (Fig. 3). The expansion of the donor cell and transconjugant populations despite competition by a large excess of plasmid-free resident recipients suggests that pCF10 provides its bacterial host with a competitive advantage in the absence of antibiotic selection or a plasmid-encoded bacteriocin (Fig. 4A and B). Due to their earlier establishment in the mice, recipient numbers at the time of introduction of the donor outnumbered the donors from 10^3^ to 10^4^ to one 5 h after donor inoculation. This ratio shifted down by the end of the experiment to approximately 80:1 recipient to donor (Fig. 4A). Similar effects were seen by enumeration from feces, where pCF10-carrying cells (donor and transconjugant) represented 0.1% of the population at the start of the experiment and expanded to approximately 10% of the total population at the end of the experiment (Fig. 4B).

### Effects of a defined microbial community on plasmid transfer.

**FIG 5:**
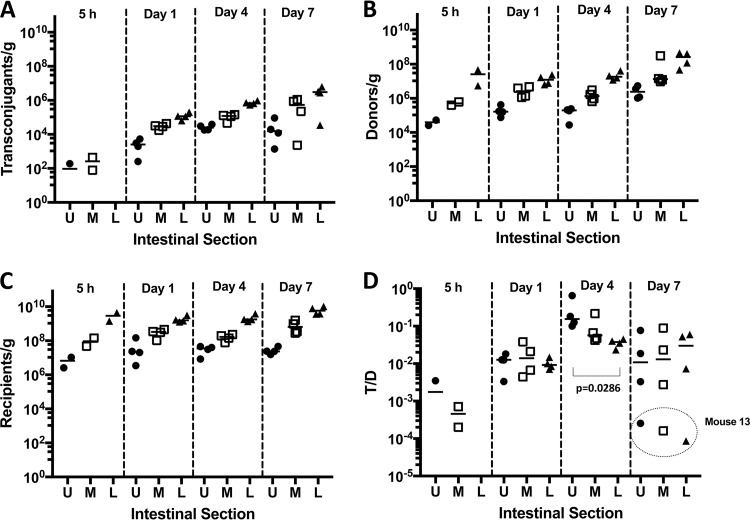
Plasmid transfer in the presence of a competing microbial community (experiment 2; Fig. 1B). (A to D) CFU/g intestinal tissue of transconjugants (A), donors (B), and recipients (C) and the transconjugant/donor (T/D) ratio (D) over time. The T/D ratio is highest on day 4 and significantly higher than in the lower intestinal tract. Mouse 13 did not show donors or transconjugants in feces on day 1.Each symbol represents the value for a section from an individual mouse, and the bars represent the median values for the intestinal sections. The upper (U) (●), middle (M) (□), and lower (L) (▲) sections of the intestinal tract are shown.

Experiment 2 investigated the effect of competing microflora on plasmid transfer; mice colonized with cCF10-producing strains (CF+) were inoculated with a defined community (*Bacteroides ovatus*, *Bacteroides vulgaris*, *Clostridium sporogenes*, *Akkermansia muciniphila*, *Faecalibacterum prausnitzii*, and *Roseburia faecis*) 3 days before introduction of the donor (Fig. 5).

**FIG 6:**
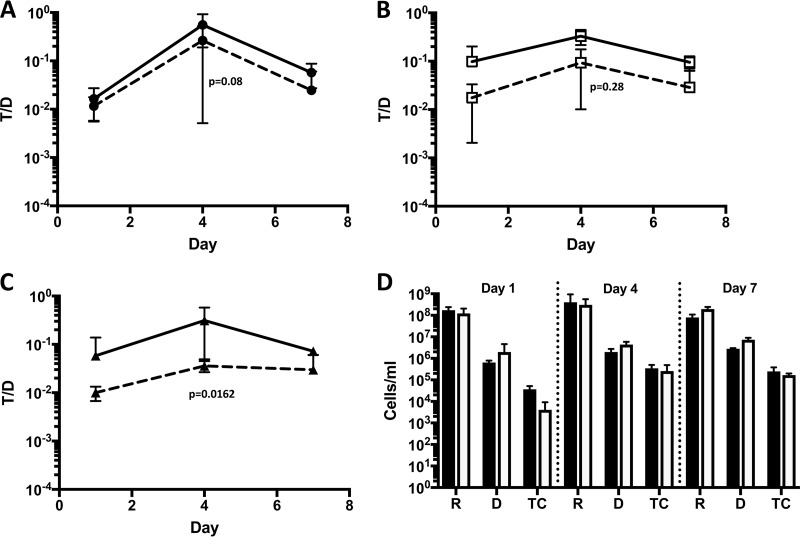
Comparison of pCF10 plasmid transfer in monospecies- and multispecies-colonized mice (experiments 1 and 2; Fig. 1B). (A to C) The plasmid transfer ratio is represented as the transconjugant/donor (T/D) ratio in the upper (A), middle (B), and lower (C) sections of the intestinal tracts of mice. T/D ratios in *E. faecalis* monospecies-colonized mice (experiment 1) (solid black lines) and in the presence of a defined community in mice (experiment 2) (broken lines) are shown. In the lower intestinal tract, the T/D ratio with the defined community present is significantly lower in comparison to the monospecies-colonized mice on day 4 (*P* = 0.0162). (D) Comparison of absolute cell numbers in the fecal material from recipient (R), donor (D), and transconjugant (TC) mice. The mice were infected with one species (black bars) or with multispecies (white bars). Values are means plus standard deviations (error bars) from two experiments.

Five hours following the introduction of donors into mice precolonized with both recipients and a defined community, transconjugants were recovered from the upper intestinal tract from only one of two mice and from the lower intestinal tract from none of the two mice. All mice sacrificed 1 day after donor addition harbored high levels of transconjugants (T/D ratio of 10^−2^ in all intestinal sections) with a peak T/D ratio (10^−1^) reached in the upper intestinal tract at day 4 and a slight decrease at day 7 (Fig. 5D). Transfer in the upper intestine appeared to be slightly more efficient, but T/D were only significantly higher compared to the lower intestinal tract (*P* = 0.0286 for the upper/lower sections; *P* = 0.2 for the upper/middle sections). A comparison of T/D ratios from experiments with and without a competing community is shown in Fig. 6A to C. The T/D ratio on day 4 was more affected by the community in the lower intestine (*P* = 0.0162), with no significant impact in the upper and middle intestinal sections (*P* = 0.08 and *P* = 0.28, respectively). Modest interference of the competing flora with plasmid transfer efficiency was also observed in the feces (*P* = 0.02). A comparison of the overall cell numbers of recipients, donors, and transconjugants in both experiments shows that the total enterococcal population densities were barely affected (Fig. 6D). Analysis of the microbial community in feces by 16S rRNA gene sequencing revealed that two members of the introduced competing community (*F. prausnitzii* and *R. faecis*) did not establish in our system. At the end of the experiment, *A. muciniphila* comprised 30% of the total population, while *B. ovatus*, *B. vulgatus*, and *E. faecalis* each comprised about a fifth of the total population. *C. sporogenes* was present at approximately 3%. Over the course of the experiment, the number of *C. sporogenes* showed the least variation, suggesting a well-defined niche (Fig. S1 and S2). *F. prausnitzii* and *R. faecis*, the least aerotolerant species studied, were not detected in the communities, suggesting a possible loss of viability during preparation of the initial inoculum or gavage.

10.1128/mBio.00037-18.1 FIG S1  Species distribution in feces from four individual mice in experiment 3. The distribution of species in the microbial community in feces in experiment 3 was determined by Illumina MiSeq sequencing of the V4 region of the 16S rRNA. The results show the results of analysis for the group of mice sacrificed on day 7 of experiment 3 (Fig. 1B). Download FIG S1, TIF file, 0.7 MB. Copyright © 2018 Hirt et al. 2018 Hirt et al. This content is distributed under the terms of the Creative Commons Attribution 4.0 International license.

10.1128/mBio.00037-18.2 FIG S2  Stability of the community in experiment 3. The percentage of the population of the recovered species is shown over time, with the data of mice 11 to 14 supplemented with data from mice sacrificed at earlier time points in the experiment. Download FIG S2, TIF file, 0.6 MB. Copyright © 2018 Hirt et al. 2018 Hirt et al. This content is distributed under the terms of the Creative Commons Attribution 4.0 International license.

### Effects of cCF10 production by recipients on plasmid transfer in the intestinal tract suggest that pheromone signaling is highly localized *in vivo.*

**FIG 7:**
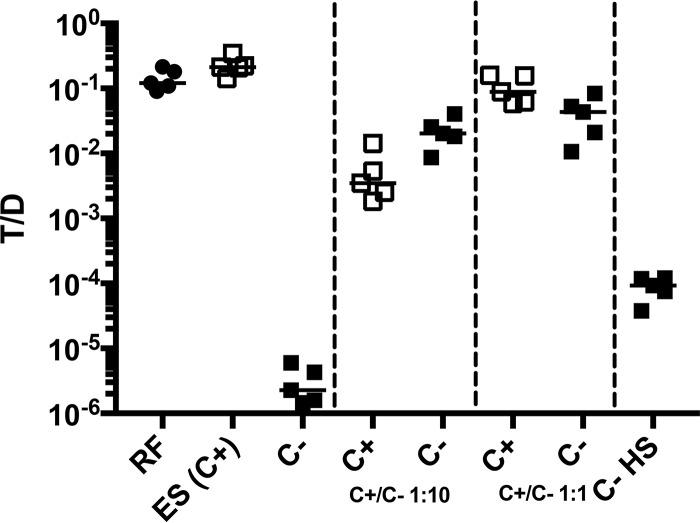
*In vitro* mating behavior of CF− versus CF+ recipients. Recipient and donor were mixed 10:1 in Todd-Hewitt broth (THB) and allowed to grow for 4 h. Samples were removed and plated on selective plates to enumerate transconjugants, donors, and recipients. The frequency of plasmid transfer from five biological replicates is shown as the transconjugant/donor (T/D) ratio. The two middle panels show the results of matings where mixtures of CF+ (C+) (□) and CF− (C−) (■) recipients were used at the ratios indicated. Results of mating performed with the CF− recipient in the presence of 10% human serum in the medium (C-HS). The commonly used recipient strain OG1RF (C+) (●) is shown for comparison.

Figure 7 shows a very high level of pCF10 transfer into two isogenic pheromone-producing strains (CF+) with different selective antibiotic resistance markers in 4-h broth matings *in vitro*. In contrast, an isogenic recipient with an engineered mutation resulting in production of a V2A biologically inactive variant of mature cCF10 (CF−), acquired pCF10 at <1/10,000 the wild-type level. Transfer into the CF− recipient strain was substantially rescued during *in vitro* matings containing mixtures of CF+ and CF− recipients if the CF+ strain comprised at least 10% of the total recipient population. A partial rescue of the CF− phenotype was also obtained by the addition of 10% human serum to the mating mixture, increasing the T/D ratio to 10^−4^ (Fig. 7).

**FIG 8:**
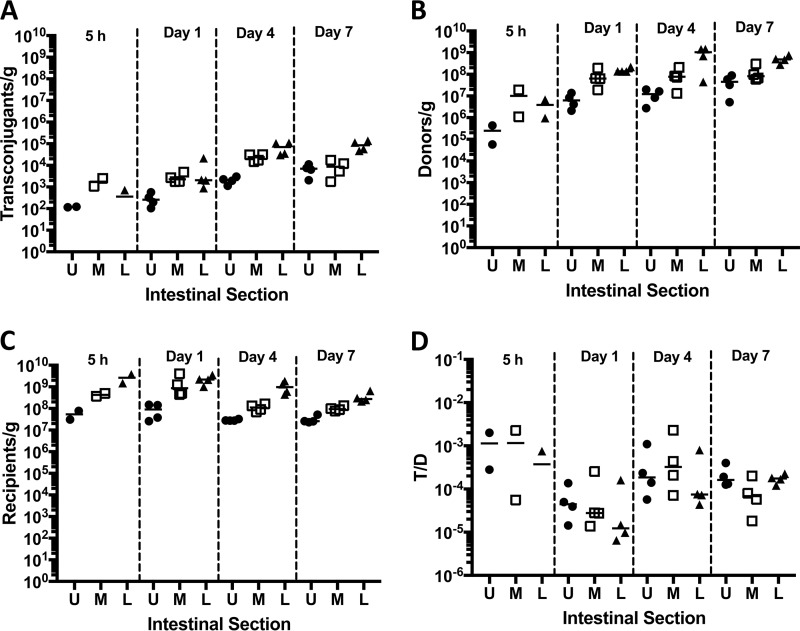
*In vivo* plasmid transfer to a CF− recipient (experiment 3; Fig. 1B). The CF− strain incapable of producing active cCF10 serves as the recipient. Four days after donor inoculation, a CF**+** recipient was introduced. (A to C) CF− transconjugant (A), donor (B), and CF− recipient (C) cell numbers over time. (D) Plasmid transfer is expressed as the ratio of CF− transconjugants/donor (T/D). The upper (U) (●), middle (M) (□), and lower (L) (▲) sections of the intestinal tract are shown. Additional results from this experiment are described in Fig. 9.

To compare the effects of pheromone production by recipients in the intestine to those observed *in vitro* (Fig. 7), we carried out experiment 3, which also examined the ability of a CF+ recipient to increase transfer into a CF− recipient by extracellular complementation. Transconjugants were recovered as early as 5 h after introduction of the donor (Fig. 8A), although the numbers were lower compared to the wild-type recipient (Fig. 2A) (*P* = 0.0286 by Mann-Whitney test for all intestinal sections). In two mice sacrificed 5 h after the addition of donors, the T/D ratio was 10^−3^ (Fig. 8A), about 10- to 100-fold lower than the ratio with CF+ donors (Fig. 2A). For the reminder of the experiment, the T/D ratio was highest on day 4 but without an apparent preference in location (*P* = 0.7984 for upper/middle sections; *P* = 0.0535 for upper/lower sections). Although the numbers of transconjugants increased over time as in experiment 1, the T/D ratio remained in the range of 10^−5^ to 10^−4^. Levels of plasmid transfer into the CF− recipient were substantially lower at all time points; for example, both total transconjugants and transconjugants/donor values were reduced by 2 to 3 orders of magnitude on day 4, which showed the highest overall frequencies with either recipient (Fig. 2A and D versus Fig. 8A and D). Results of the *in vivo* experiment depicted in Fig. 8A and D indicate higher frequencies of transfer (10- to 100-fold) into the CF− recipient in the intestine than in the *in vitro* experiment (Fig. 7). This is likely due to host factor-mediated low-level induction of donors in the intestinal tract, as observed *in vitro* in the presence of serum (Fig. 7), although other mechanisms are also possible.

**FIG 9:**
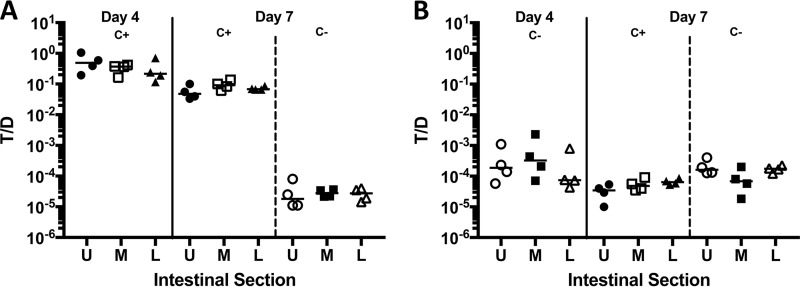
Effect of the order of addition of the alternate recipient strain (experiments 1 and 3; Fig. 1B). (A) T/D ratio with CF+ (c+) as the original recipient before (day 4) and after (day 7) the addition of the CF− (c−) recipient. (B) T/D ratio with CF− as the original recipient before (day 4) and after (day 7) the addition of the C+ recipient. The upper (U) (●), middle (M) (□), and lower (L) (▲) sections of the intestinal tract are shown.

To test whether the presence of pheromone-producing recipients in the intestinal tract could increase plasmid transfer into coresident pheromone-less recipients, as observed *in vitro* (Fig. 7), we gavaged some mice from experiments 1 and 3 that had harbored both a recipient strain (CF+ in experiment 1 and CF− in experiment 3) and a wild-type donor strain for 4 days with a differentially marked recipient with the opposite pheromone production phenotype. In both experiments, the recipient added at day 4 never achieved population densities more than 2.5% (upper intestinal tract, experiment 1) of the total recipient population at day 7, while averaging 0.75% ± 0.17% in all other sections and animals. Likewise, transfer into the CF− recipient was much less frequent than into the wild type when transconjugants/recipient were determined (Fig. 9A and B). In both experiments, plasmid transfer into the CF− recipient was not rescued by the presence of the CF+ strain, regardless of whether the CF− recipient was added before or after the CF+ recipient. This suggests that in the GI tract, effective pheromone signaling is restricted to donor and recipient cells in close proximity. Since we have previously shown production of microcolony biofilms on the mucosal surface throughout the length of the intestine (13), it is tempting to speculate that these adherent communities could be important ecologic niches for pheromone signaling and plasmid transfer *in vivo*.

## DISCUSSION

The occurrence of highly efficient transfer of the *E. faecalis* pheromone-responsive plasmid pCF10 in a germfree mouse model was expected, since high transfer ratios had been reported in several mammalian model systems before (10–12). However, the *in vivo* role of pheromones in plasmid transfer has not been examined. We also note that all previous studies of enterococcal plasmid transfer in the GI tract used either animals treated with antibiotics, plasmids carrying genes that encoded markers that could select against recipients during the course of the experiment, or both. These factors limit the ability to determine the intrinsic efficiency of transfer. The use of a germfree model allowed us to examine pheromone signaling and plasmid transfer under defined conditions without antibiotic selection and to begin to assess how multispecies communities impact this process. We detected high-level plasmid transfer in the intestinal tract 5 h after inoculation of the donor, the earliest time point thus far reported for transfer *in vivo*. The fact that transconjugants and donors could not be detected 5 h after donor inoculation in feces strongly supports the hypothesis that plasmid transfer occurs in the intestinal tract and is not a result of extracorporeal plasmid transfer in the feces followed by coprophagy. Plasmid transfer was detected in all three experiments in five of six mice in the upper intestinal tract, all six mice in the middle section, and two of six mice in the lower intestinal tract. These observations suggest that the upper and middle sections of the intestinal tract are favorable, if not preferred, locations for transfer of pCF10. Lack of detection of early plasmid transfer in the lower intestinal tract in the mice with the competing microflora supports the view that the competing effect of the defined intestinal community is stronger in the environment with the greatest microbial density.

Plasmids are often considered to be a metabolic burden for the cell that may be selected for under certain conditions, such as the presence of an antibiotic. Interestingly, the percentage of plasmid-carrying cells (donors plus transconjugants) in the total enterococcal population increased during the experiments reported here and in preliminary experiments (data not shown). This suggests that pCF10 provides one or more factors that confer a selective advantage to plasmid-carrying cells in the absence of antibiotic or bacteriocin selection. A candidate for this selective advantage is aggregation substance (AS), encoded by the *prgB* gene on pCF10. A previous study using an endocarditis model showed that a strain carrying a pCF10 derivative deleted of *prgB* was dramatically attenuated relative to both wild-type pCF10-carrying cells and to isogenic plasmid-free cells, suggesting that the *prgB*-free plasmid comprised a burden to the host strain (14). The selective advantage provided by AS in the endocarditis model has been attributed to the increased resistance to polymorphonuclear leukocyte (PMN)-mediated killing (15) provided by two RGD sequences in the protein and to increased colonization ability (14, 16). The presence of AS could also provide a selective advantage for the donor in the intestinal tract. It is conceivable that AS allows the donors to expand the ecologic niche for *E. faecalis* in the intestinal tract by serving as an adhesin to host cells (17, 18) and mucosal proteins and by attaching to the already established recipient microcolonies on the intestinal wall (13) with subsequent plasmid transfer. Aggregation substance is not the only gene product that might affect fitness. Since pCF10 carries many other genes (19), including *uvr* orthologs, and genes encoding numerous secreted cell envelope proteins that could impact competitive fitness in the GI tract, we are currently examining this issue comprehensively using a transposon sequencing approach. The introduction of a defined community after the recipient was inoculated but before the donor was administered had little effect on plasmid transfer in the upper and middle sections of the intestine but modestly reduced transfer in the lower intestinal tract. This might be expected, since the members of the defined community are normal inhabitants of the large intestine, and thus, it is likely that the reduced T/D rate is due to spatial interference with the interaction between donor and recipient. *E. faecalis* still comprised about 20% of the community in the large intestine based on 16S rRNA gene sequencing of the feces, consistent with the only marginally reduced numbers of enterococci recovered in comparison to the monospecies-colonized mice.

A major aspect of pheromone signaling in *E. faecalis* that has not been addressed is the role of the conjugation-inducing pheromones such as cCF10 *in vivo*. Licht et al. showed that cCF10 had biological activity in intestinal mucus *in vitro* (20); the high plasmid transfer rates in the previously described *in vivo* experiments would suggest that cCF10 induction was active under *in vivo* conditions, but direct evidence for the role of cCF10 had not been obtained. We used a strain deficient in the production of a functional cCF10 peptide as a recipient to investigate contributions of the peptide *in vivo*. With the mean T/D ratio 10^2^- to 10^3^-fold lower compared to the CF+ recipient (day 4), the results from experiment 3 demonstrated the requirement of cCF10 for high-frequency plasmid transfer *in vivo*. However, plasmid transfer to the CF− recipient still occurred with a T/D ratio of 10^−4^, which was considerably higher than that observed *in vitro* with the same donor and recipient (Fig. 7). The observed level of plasmid transfer with a CF− recipient *in vivo* is most likely due to the previously described autoinduction of the donor by the donor cell’s own cCF10 (9). Autoinduction is triggered by sequestration of the inhibitory peptide iCF10 by a host factor, possibly albumin/lipid complexes (9). We could indeed show that the addition of serum *in vitro* could partially restore plasmid transfer to the CF− recipient (Fig. 7). Currently, the identity of a host factor(s) that would cause donor induction in the intestinal tract is not known and will be the focus of future studies. Our attempt to restore efficient plasmid transfer to the CF− strain by addition of the CF+ wild-type recipient to the mouse GI tract did not significantly increase the T/D ratio for the CF− recipient. This was most clearly demonstrated when the CF+ recipient was established first and CF− was added at day 4, with a CF+**/**CF− ratio between 40 and 150:1 (experiment 1; Fig. 9A). This result indicates that recipient-produced cCF10 is not freely available throughout the intestine for donor induction. Plasmid transfer to CF− recipients could be initiated by autoinduction of the donor with subsequent expression of AS and binding to the resident recipient microcolonies. Subsequent plasmid transfer within the colony could be dependent on the production of CF by the other recipients in the colony. A recent study in germfree mice with a defined community indicated low bacterial density in the lumen of the intestinal tract particularly in the small intestine (21), suggesting that donors and recipients in our study may be too far apart for direct cell-cell communication in the lumen. In addition, these investigators showed that even in the large intestine, bacterial cells are concentrated on the intestinal wall (20). This would support our hypothesis that plasmid transfer most likely occurs in established microcolonies.

In all three experiments, plasmid transfer occurred at a high level in the intestinal tract as early as 5 h after donor inoculation, and before either donors or transconjugants could be detected in feces. In feces, where individual mice could be monitored over time, the rates of increase of recipients and donors between day 1 and day 4 were remarkably similar in all three experiments (see Fig. S3A in the supplemental material). Interestingly, while the number of recipients showed a tendency to decrease following donor colonization in experiments 1 and 3, the presence of the competing community appeared to abrogate this effect. The spread in rates was wider for transconjugants, also reflected in T/D ratio rates, indicating variability of plasmid transfer and proliferation in the recipient population between individual mice (Fig. S3B). The rate of increase of transconjugants or T/D ratio between day 1 and day 4 was, however, not significantly different in the three experiments. The influence of spatial structure in restricting plasmid transfer in biofilms has been observed before (22), and our results indicate that a similar mechanism could be occurring here. In samples enumerated directly from the intestinal tract, where T/D ratios were generally higher than in feces, the number of samples available did not provide sufficient statistical power for definitive conclusions, but the average T/D ratios on day 4 compared to the T/D ratios on day 1 suggested that the upper intestinal tract could be an equally, if not more favorable, niche than the lower intestine for pheromone-inducible plasmid transfer.

10.1128/mBio.00037-18.3 FIG S3  Rate of increase of *E. faecalis* strains in feces from day 1 to day 4. (A) Fold increase/decrease of recipients (R) and donors (D) from day 1 to day 4. (B) Fold increase/decrease of transconjugants (TC) and the transconjugant/donor (T/D) ratio from day 1 to day 4. Download FIG S3, TIF file, 0.4 MB. Copyright © 2018 Hirt et al. 2018 Hirt et al. This content is distributed under the terms of the Creative Commons Attribution 4.0 International license.

In summary, we have shown that pCF10 plasmid transfer occurs within 5 h of donor introduction in a germfree mouse model in the intestinal tract. Early sampling and results from the day 4 time points suggest that the upper intestinal tract provides the most favorable environment for plasmid transfer. The introduction of a simple multispecies community did not substantially impact *E. faecalis* cell numbers and plasmid transfer rate, except in the lower intestinal tract. The failure to complement the pheromone-negative strain with a wild-type recipient suggests the need for close contact between recipient and donor *in vivo*. These studies represent the initial investigation into how plasmid carriage and cell-cell signaling may impact the ecology of *E. faecalis* in the GI tract. The ability to introduce a variety of genetic constructs into this well-defined model and to vary the overall complexity of the community will ultimately enhance our understanding of important questions related to how antibiotics and other inducers of GI tract dysbiosis impact the transition of the enterococci from harmless commensal organisms to lethal nosocomial pathogens.

## MATERIALS AND METHODS

### Bacterial strains.

*In vivo* transfer experiments employed either a CF+ wild-type recipient OG1RF (23) and OG1ES (24) or an isogenic CF− strain producing the peptide LATLVFV rather than the native CF peptide LVTLVFV (Chandler et al. [9]). The *Enterococcus faecalis* plasmid donor for all experiments was the wild-type strain OG1Sp(pCF10) (C. J. Kristich, unpublished). Stocks of *E. faecalis* were made and frozen at −80°C; isolates were grown from stocks before each experiment. A defined community of *Bacteroides ovatus*, *Bacteroides vulgatus*, *Clostridium sporogenes*, *Akkermansia muciniphila* ATCC BAA-835, *Faecalibacterum prausnitzii* ATCC 27755, and *Roseburia faecis* DSMZ 16840 was used as competing GI microflora. For these bacteria, stock solutions were made of each strain in prereduced medium inside an anaerobic chamber and frozen at −80°C.

### Selective plates.

CF+ and CF− recipients were grown and enumerated on tryptic soy agar (TSA) plates with 50 µg/ml rifampin, OG1ES on TSA with 10 µg/ml erythromycin, and OG1Sp(pCF10) on TSA with 630 µg/ml spectinomycin plus 10 µg/ml tetracycline. Transconjugants were selected with 50 µg/ml rifampin plus 10 µg/ml tetracycline for OG1RF(pCF10) and 10 µg/ml erythromycin plus 10 µg/ml tetracycline for OG1ES(pCF10).

### Gnotobiotic mice.

All animal experiments were approved by the institutional IACUCs at both the Mayo Clinic, Rochester, MN (25, 26), and at the University of Minnesota, with the latter giving final overall approval of the experiments (protocol 1511-33206A). Gnotobiotic experiments were done using germfree (GF) 5- to 6-week-old Swiss Webster mice (male and female) maintained and bred in sterile isolators (Class Biologically Clean, Ltd., Madison, WI) at the Mayo Clinic gnotobiotic facility. GF status of isolators and mice was confirmed using a combination of culture (Sabouraud dextrose medium, brain heart infusion medium, and nutrient broth medium at 37°C for 7 days under aerobic and anaerobic conditions) and PCR (using universal [27, 28] and genus-specific primers [29] to screen for bacterial DNA encoding 16S rRNAs prior to initiating experiments). Mice were fed an autoclaved standard diet (Purina LabDiet 5K67). Individual mouse identity was tracked using ear punches and cage number.

### Mouse inoculation for *in vivo* transfer experiments.

The germfree mice described above were used to examine pCF10 transfer in the intestinal tract; for the experiments described in this paper, recipients were first introduced, followed by donors (Fig. 1B). For experiment 1, mice received 4.3 × 10^7^ CFU of wild-type recipient OG1ES/mouse on day −3 and 2.9 × 10^7^ CFU of mutant CF− recipient (OG1RF background)/mouse on day 4 (Fig. 1B). For experiment 2, mice received 6.3 × 10^7^ CFU of mutant CF− recipient (OG1RF background)/mouse on day −3 and 1.0 × 10^7^ CFU of wild-type recipient OG1ES/mouse on day 4 (Fig. 1B). For experiment 3, mice received 4.3 × 10^7^ CFU of wild-type recipient OG1ES/mouse on day −4, and the defined community as competing GI microflora was gavaged on day −3 (Fig. 1B). For the defined community gavage, immediately after thawing the stocks, 350 µl of each stock was mixed, and 100 µl of the mixture was gavaged into the mice within 30 min. Mice received oral gavage with 2.0 × 10^8^ to 4.4 × 10^8^ CFU of *E. faecalis* plasmid donors/mouse on day 0 for all experiments (Fig. 1B).

### Enumeration of donors, recipients, and transconjugants.

Fourteen mice were used for each of three experiments; donors, recipients, and transconjugants were enumerated from fecal samples on days 1, 4, and 7 and from GI tissue (an upper section, a middle section, and a lower section) at 5 h (*n* = 2) and at 1 (*n* = 4), 4 (*n* = 4), and 7 (*n* = 4) days by selective plate quantitative culture. The upper GI section includes the duodenum plus the proximal jejunum, the middle GI section includes the distal jejunum and the ileum, and the lower GI section consists of the colon. Fecal samples were collected from individual mice and placed in 1 ml sterile saline, homogenized, quantitatively cultured, and reported as log_10_ CFU/milliliter. The GI tissue sections were aseptically dissected, and the fecal contents were removed, sliced open longitudinally, weighed, and placed into 1 ml sterile saline. They were vortexed for 30 s, sonicated for 5 min (frequency, 40 ± 2 kHz; power density, 0.22 ± 0.04 W/cm^2^; Zenith Ultrasonic Inc., Norwood, NJ), and vortexed an additional 30 s. The sonicate fluid was quantitatively cultured, and log_10_ CFU/gram of tissue was calculated. The frequency of plasmid transfer is reported as transconjugant/donor (T/D) ratio.

### *In vitro* plasmid transfer.

Recipients were inoculated 1:10 and donors were inoculated 1:100 in 2 ml of fresh Todd-Hewitt broth (Becton, Dickinson and Company, Sparks, MD). Plasmid transfer was assessed after 4 h coculture of donor and recipients by enumeration of donors and transconjugants by plating on selective plates as described above.

### Characterization of the microbial community.

The defined microbial community used was characterized by partial 16S rRNA gene sequencing of the communities found on days 1, 4, and 7 in mouse feces and also in an aliquot of the original gavage input. Fecal DNA was isolated by previously described methods (30, 31). Library generation and sequencing were performed at the University of Minnesota Genomics Center. The V4 region of 16S rRNA gene was amplified, and 100,000 reads/sample were performed on an Illumina MiSeq platform with two 300-bp paired-end reads.

### Statistical analysis.

Analysis was performed by the in-program suite of the Prism7 software package (GraphPad Software, Inc.).
