# Supplementary material for: Enterococcus faecalis Sex Pheromone cCF10 Enhances Conjugative Plasmid Transfer In Vivo
Source: mBio. 2018 Feb 13;9(1):e00037-18. doi: 10.1128/mBio.00037-18 (PMC5821081; doi:10.1128/mBio.00037-18)
Supplement: TABLE S1 [file mbo001183729st1.docx]

|  | **5 h** | **24 h** |
| --- | --- | --- |
| Recipients | 1.63E+08 ± 8.2E+07 | 3.92E+07 ± 8.7E+06 |
| Donors | 0 | 1.70E+06 ± 8.4E+05 |
| Transconjugants | 0 | 3.94E+04 ± 2.8E+04 |
| T/D | 0 | 2.81E-02 ± 0.021 |

Table S1:

Number of recipients, donors and transconjugants in feces. Preliminary experiment. Donor: OG1Sp:pCF10, Recipient: OG1RF. Fecal samples were obtained from 12 mice.
