# Supplementary material for: Enterococcus faecalis Sex Pheromone cCF10 Enhances Conjugative Plasmid Transfer In Vivo
Source: mBio. 2018 Feb 13;9(1):e00037-18. doi: 10.1128/mBio.00037-18 (PMC5821081; doi:10.1128/mBio.00037-18)
Supplement: TABLE S2 [file mbo001183729st2.docx]

|  | **C+** | **C-** |
| --- | --- | --- |
| **Day 2** |  |  |
| Upper | 3.35E-02 ± 0.023 | 7.30E-04 ± 0.0002 |
| Middle | 8.46E-02 ± 0.059 | 7.28E-04 ± 0.0007 |
| Lower | 3.62E-02 ± 0.037 | 1.39E-03 ± 0.0011 |
| Feces | 2.79E-02 ± 0.007 | 2.30E-05 ± 2.02E-05 |
| **Day 4** |  |  |
| Upper | 6.82E-01 ± 0.368 | 3.81E-04 ± 0.0002 |
| Middle | 8.50E-02 ± 0.061 | 3.51E-04 ± 0.0002 |
| Lower | 5.95E-02 ± 0.017 | 9.92E-05 ± 5.18E-05 |
| Feces | 1.35E-01 ± 0.11 | 3.16E-04 ± 0.0002 |
| **Day 7** |  |  |
| Upper | 6.62E-02 ± 0.013 | 4.68E-03 ± 0.0034 |
| Middle | 6.09E-02 ± 0.015 | 2.60E-03 ± 0.0028 |
| Lower | 3.54E-02 ± 0.030 | 1.29E-02 ± 0.0093 |
| Feces | 6.37E-02 ± 0.018 | 4.15E-03 ± 0.0013 |

Table S2: T/D rates in two preliminary experiments with wild-type (C+) and cCF10 deficient mutant (C-) as established recipient in the germ-free mouse model. In the C- experiment, C+ recipient is introduced to the mice on day 4. Average of 4 mice and standard deviation. Upper, Middle, Lower: Intestinal section
