# Supplementary figures and images for: Enterococcus faecalis Sex Pheromone cCF10 Enhances Conjugative Plasmid Transfer In Vivo
Source: mBio. 2018 Feb 13;9(1):e00037-18. doi: 10.1128/mBio.00037-18 (PMC5821081; doi:10.1128/mBio.00037-18)

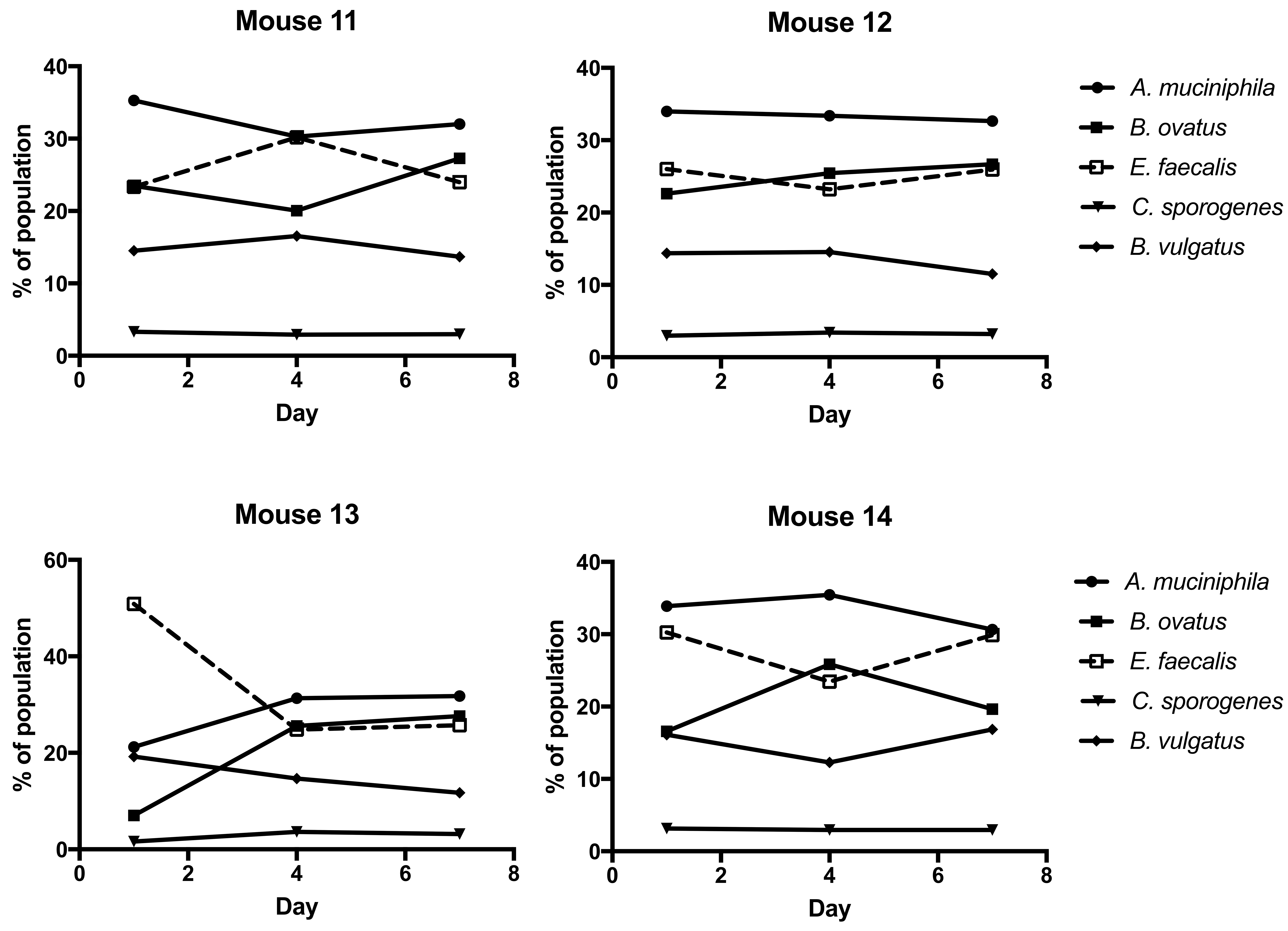

Supplement: FIG S1 [file mbo001183729sf1.tif]

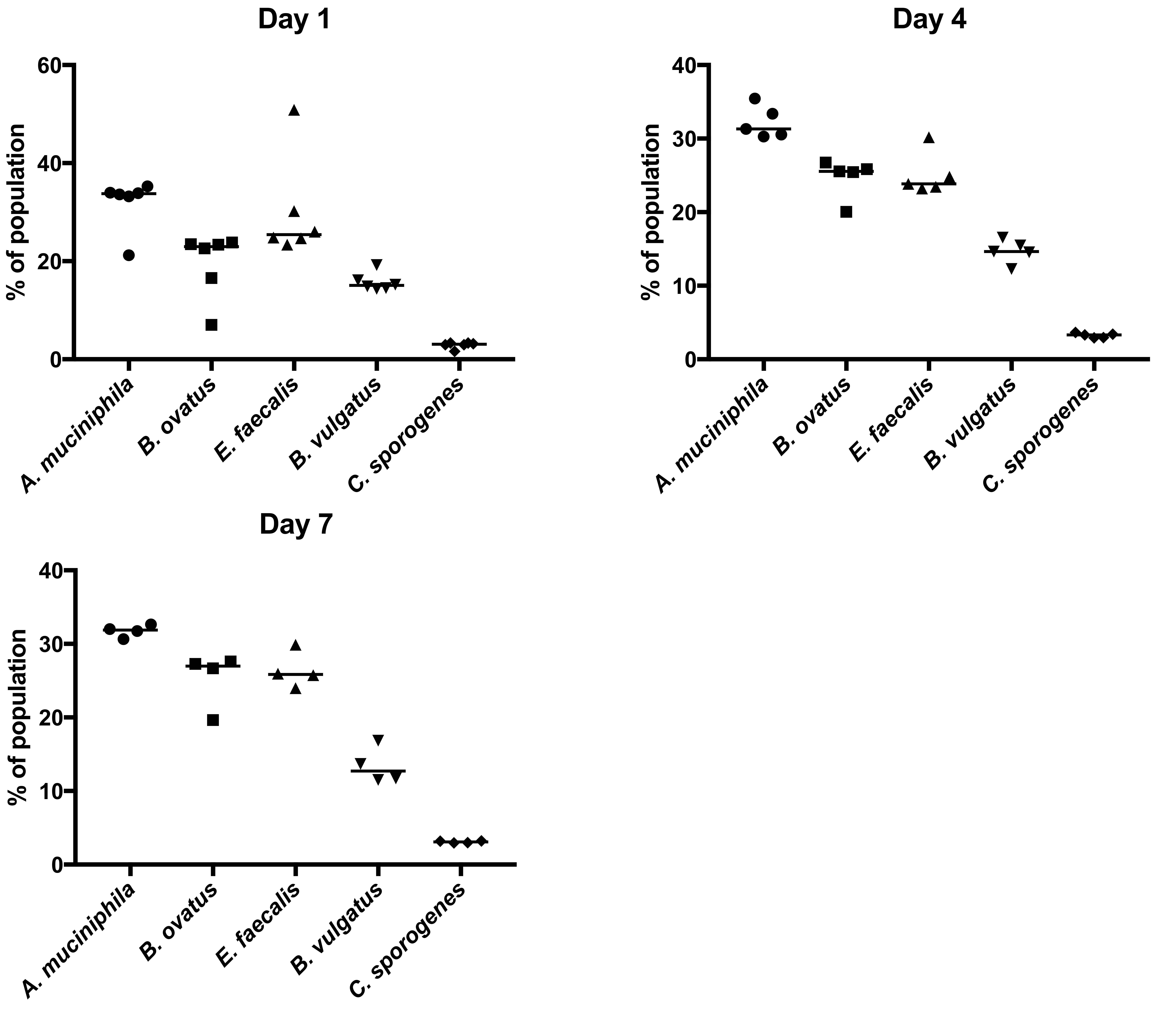

Supplement: FIG S2 [file mbo001183729sf2.tif]

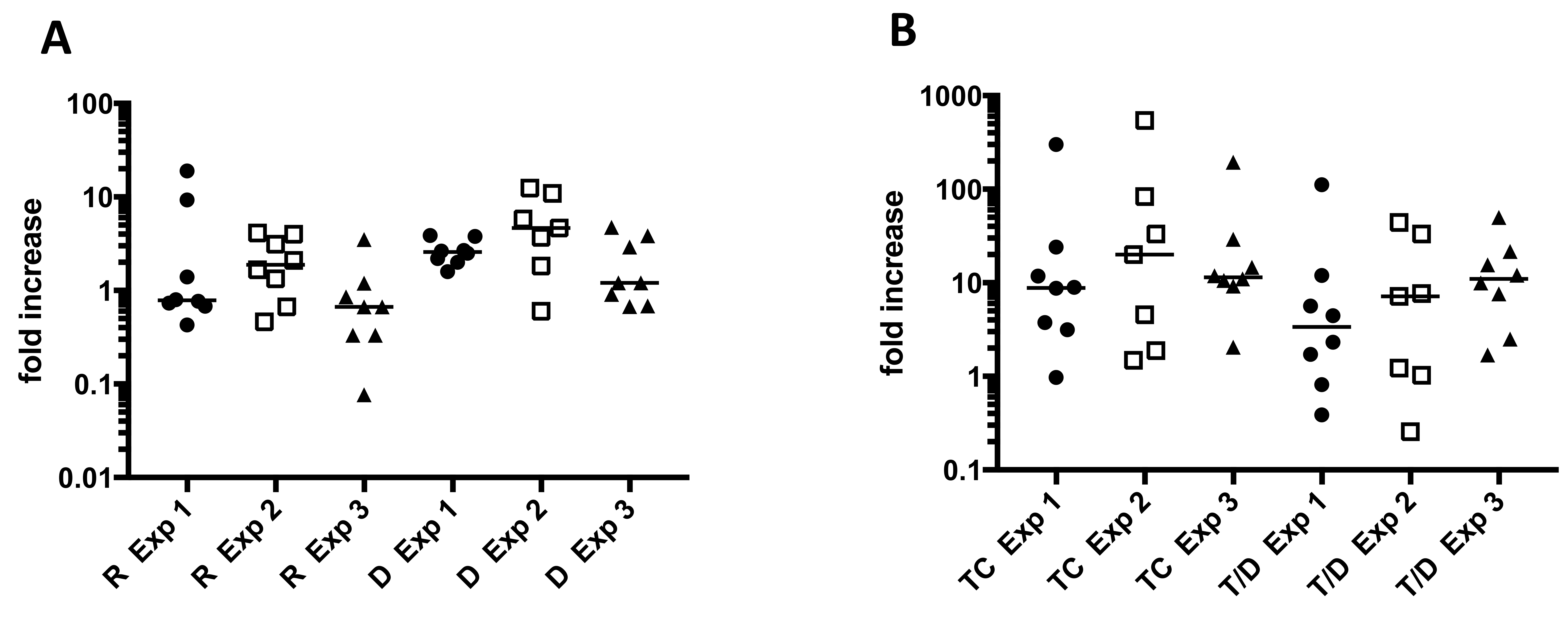

Supplement: FIG S3 [file mbo001183729sf3.tif]
